# Obstructive Dysphagia and Positional Dyspnea: Can You Identify the Cause?

**Published:** 2018-05-01

**Authors:** Kimberly Dotter

**Affiliations:** Anderson Cancer Center

Mr. V is a 25-year-old man with no significant past medical history who began experiencing shortness of breath while lying supine, along with changes in swallowing over the past several months. More recently, he noted a neck mass while shaving. Mr. V presented to clinic with complaints of a nontender left-sided neck mass, positional dyspnea, and general neck pressure when lying on his right side. He also reports a sensation of food sticking after it is swallowed, particularly with solids as opposed to liquids. He localizes the sensation of dysphagia in the region of the suprasternal notch that has slowly become more noticeable over time. He has no associated symptoms of chest pain, heartburn, pain with swallowing, or unintentional weight loss. He denies any radiation exposure to the head and neck area or previous neck surgery. He has no preexisting medical conditions such as diabetes, history of stroke, cancer, or heart or lung disease, and takes no medications. There is no significant family history of cancer. He is a nonsmoker, does not drink alcohol, and denies illicit drug use.

## SYSTEMS AND PHYSICAL EXAM

He endorses fatigue, but attributes this to his extensive work hours. He denies fever, chills, or diaphoresis. He denies any bruising or night sweats. He has positional shortness of breath as described above, but he has no hoarseness and denies any choking or coughing episodes.

Mr. V is a healthy-appearing male in no acute distress. He appears well-developed and well-nourished. His vital signs are within normal limits. He is oriented to person, place, and time. There are no cranial nerve deficits.

His head is normocephalic and atraumatic. The oropharynx is clear and moist without obvious lesions. Conjunctivae are normal and extraocular muscles are intact and full. Pupils are equal, round, and reactive to light. He does not have scleral icterus. His neck has a normal range of motion and is supple. There is no tracheal deviation present on inspection or sign of prior neck surgery. A left-sided fullness is noted on observation. On palpation, there are multiple thyroid nodules in the bilateral lobes that are mobile and move well with deglutition. The left-sided mass is greater than 5 cm with inferior borders difficult to palpate. His voice is clear on phonation and swallowing is intact. He has no palpable cervical or supraclavicular adenopathy. All other aspects of physical examination are unremarkable or within normal limits.

**Figure 2:**
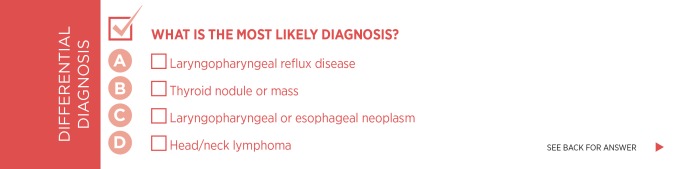
What is the most likely diagnosis?

## CORRECT ANSWER: B

**Diagnostic Imaging**

The baseline neck ultrasound reveals:

1. Multinodular thyroid gland with significantly enlarged left lobe.
2. Ultrasound-guided fine-needle aspiration (FNA) of dominant right thyroid lobe nodule measuring 2.2 × 1.4 × 2 cm is consistent with Hürthle cell lesion (indeterminate diagnostic category).
3. Ultrasound-guided fine-needle aspiration of left thyroid complex mass measuring 5.4 × 3.7 × 4.8 cm is consistent with Hürthle cell lesion (indeterminate diagnostic category).
4. No adenopathy in the jugular territories or anterior neck.

**Figure 1:**
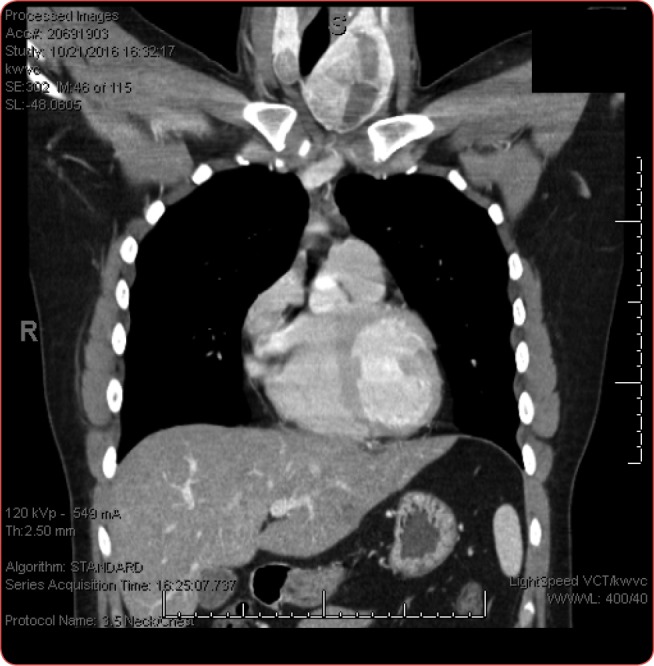
CT of head and neck.

Additional imaging also included a computed tomography of the neck and chest (see Figure 1) with confirmed large left-sided mass causing displacement of the trachea to the right, and unremarkable findings in the chest. Laboratory testing showed no abnormalities. His thyroid function, complete blood count, and Chem-7 panel were within normal limits.

**Rationale for Thyroid Nodule or Mass**

Aerodigestive compressive symptomatology is a common complaint among outpatient clinics, with presentations ranging from mild neck pressure or a globus sensation, to more severe with significant dysphagia or dyspnea (Banks et al., 2011). Dysphagia is a subjective sensation of difficulty swallowing, and if reported by patients, implies that there is an issue with either anatomical or mechanical obstruction or motor function. This sensation can be caused by a variety of different medical conditions; therefore, further diagnostic testing will depend upon the patient’s medical history and the information derived from the physical examination before moving forward with any testing to evaluate for true esophageal dysmobility (Trate, Parkman, & Fisher, 1996).

Laryngopharyngeal reflux is the most common cause of this swallowing or globus complaint, but the examination of the neck and pharynx is normal (Burns & Timon, 2007). Thyroid pathology is also common and can be a cause of compressive symptomatology in both benign and malignant disease (Nam et al., 2015). Thyroid enlargement can cause direct compression of the trachea leading to dyspnea, especially while lying supine. Dysphagia can be a direct result of compression of the esophagus by an enlarged thyroid gland or nodule. In more advanced cases, it can be caused by direct invasion or nerve involvement by a cancer (Banks et al., 2011).

Thyroid nodules, or an abnormal growth of thyroid cells that forms a lump or swelling in the thyroid gland, are a very common entity among the general population. They are usually asymptomatic and found incidentally. At least half of the population will have a thyroid nodule identified by examination or imaging by the age of 60. The prevalence of palpable thyroid nodules is approximately 5% in women and 1% in men. The clinical importance of these nodules resides in the necessity to rule out potential malignancy, which is dependent on multiple factors, including age, sex, family history, and personal history of radiation exposure, and can range from 7% to 15% of cases. Based on American Thyroid Association (ATA) guidelines, an ultrasound with FNA is the gold standard for an evaluation of thyroid nodules (Haugen et al., 2016). The incidence of thyroid carcinoma in general is low, but some studies have shown that it is higher in nodules greater than 4 cm (McCoy et al., 2007). An algorithm of the Bethesda system for reporting thyroid FNA cytopathology includes diagnostic categories and risk of malignancy (Table 1).

**Table 1:**
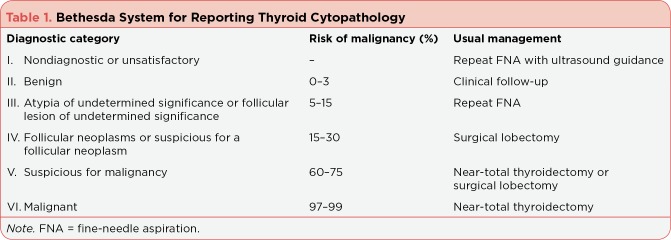
Bethesda System for Reporting Thyroid Cytopathology

Given the large size of the nodule, presence of compressive symptoms, and the indeterminate diagnosis category on Bethesda criteria (Baloch et al., 2008), the patient meets multiple criteria for surgical intervention.

Hürthle cells can be found in nodules across the spectrum of thyroid disease. Their significance is uncertain. The current diagnosis of a malignant Hürthle cell neoplasm depends on architectural findings of capsular and/or vascular invasion of the surgically resected specimen. This proves challenging in determining the appropriate course of action, as there is not a cytopathologic feature on the FNA specimen that can differentiate between a Hürthle cell hyperplasia or adenoma and a Hürthle cell neoplasm. Patient-specific clinical context, individual risk factors, and other clinical parameters will help determine preoperative decision-making and the extent of surgery. Among these risk factors are older patient age, size of tumor, and male sex, which have been reported to be more highly associated with malignancy, but reports for which have been inconsistent in the literature (Cannon, 2011). The recommendation was to proceed with surgical resection to obtain a definitive diagnosis.

## EXPLANATION OF INCORRECT ANSWERS

**Laryngopharyngeal reflux disease.** The patient denies any history of heartburn or other symptoms of reflux disease.

**Laryngopharyngeal or esophageal neoplasm.** Imaging confirms large thyroid nodules with no evidence for upper airway neoplasms or masses. This would also be an unlikely diagnosis in a young patient with no social risk factors.

**Head/neck lymphoma.** In this patient’s case, as a young male presenting with a neck mass, it is important to keep thyroid malignancy and lymphoma as possible differential diagnoses. Lymphomas are a common cause of lymphadenopathy in the head and neck and often present with an unknown mass (Urquhart & Berg, 2001). On physical examination of this patient, the large thyroid nodules in the setting of no palpable local or regional adenopathy makes a lymphoma diagnosis less likely. There is no adenopathy seen on the neck imaging. He does not exhibit any other symptoms consistent with a lymphoproliferative disorder and the FNA cytopathology also rules out this diagnosis. A primary thyroid lymphoma is a rare cancer and could be eliminated as a possibility through biopsy cytopathology and additional immunocytochemical staining if needed (Kwak et al., 2007).

## MANAGEMENT

Mr. V underwent a left thyroid lobectomy without complication, and his compressive symptomatology has resolved. The final pathology was consistent with a 6-cm follicular neoplasm of uncertain malignant potential. His case was presented at the MD Anderson Cancer Center Multidisciplinary Planning Conference. The final recommendation was to proceed with close observation and serial ultrasounds of the remaining lobe, given the patient’s young age and effort to retain thyroid function and avoid potential thyroid hormone dependency. Should there be any growth of the remaining dominant right-sided nodule in the future, we would reassess for possible completion thyroidectomy. The ATA guidelines recommend assessing thyroid function at 6 to 8 weeks after surgery, as needed with symptom changes, and yearly at minimum.

The term "thyroid tumor of uncertain malignant potential" is a gray zone subgroup of follicular-patterned thyroid tumors for which a diagnosis of benign or malignant cannot be assessed with full certainty. The diagnostic criteria used to establish a diagnosis can be subtle and subjective. The frequency, diagnostic reproducibility, and other testing such as molecular profiling of such tumors have been insufficiently explored. According to the literature, since most of these will behave in an indolent manner with a rare risk of recurrence or metastasis, this borderline or intermediate diagnosis will help to prevent unnecessary additional surgery or adjuvant therapy, such as radioactive iodine ablation (Baloch & LiVolsi, 2002).
